# Supplementary material for: Non-relativistic torque and Edelstein effect in non-collinear magnets
Source: Nat Commun. 2024 Sep 3;15:7663. doi: 10.1038/s41467-024-51565-6 (PMC11372084; doi:10.1038/s41467-024-51565-6)
Supplement: Supplementary file 1 — Supplementary Information [file 41467_2024_51565_MOESM1_ESM.pdf]

Supplementary Material: Non-relativistic torque and Edelstein effect in non-collinear magnets

Rafael González-Hernández[1], Philip Ritzinger[2], Karel Výborný[2], Jakub Železný[2], Aurélien Manchon[3]
[1]*Grupó de Investigación en Física Aplicada, Departamento de Física, Universidad del Norte, Barranquilla, Colombia;* [2] *Institute of Physics, Czech Academy of Sciences, Cukrovarnická 10, 162 00 Praha 6 Czech Republic;* [3]*Aix-Marseille Univ, CNRS, CINaM, Marseille, France*

SYMMETRY TENSORS

Here we give the general symmetry-restricted form of the Edelstein effect response tensor χ for the systems discussed in the text. In Table I we give the symmetry tensors for Mn_3Sn. In Table II we give the symmetry tensors for the Mn_3Ir [111] bilayer. The symmetry of every Mn layer is the same. We note that we have constructed the bilayer in such a way that there are in fact six Mn sublattices in each layer. However, three of those are connected to the other three by translation and are thus equivalent. In Tables III and IV we give the $\mathcal{T}$-even resp. $\mathcal{T}$-odd symmetry tensors for $LuFeO_3$.

	A	B	C
$\mathcal{T}$-even no soc	$\begin{pmatrix} 0 & 0 & 0 \\ 0 & 0 & 0 \\ \sigma^A_{zx} & -\sqrt{3}\sigma^A_{zx} & 0 \end{pmatrix}$	$\begin{pmatrix} 0 & 0 & 0 \\ 0 & 0 & 0 \\ \sigma^A_{zx} & \sqrt{3}\sigma^A_{zx} & 0 \end{pmatrix}$	$\begin{pmatrix} 0 & 0 & 0 \\ 0 & 0 & 0 \\ \sigma^A_{zx} & \sqrt{3}\sigma^A_{zx} & 0 \end{pmatrix}$
$\mathcal{T}$-even soc	$\begin{pmatrix} 0 & 0 & \sigma^A_{xz} \\ 0 & 0 & -\sqrt{3}\sigma^A_{xz} \\ \sigma^A_{zx} & -\sqrt{3}\sigma^A_{zx} & 0 \end{pmatrix}$	$\begin{pmatrix} 0 & 0 & \sigma^B_{xz} \\ 0 & 0 & \sigma^B_{yz} \\ \sigma^B_{zx} & \sigma^B_{zy} & 0 \end{pmatrix}$	$\begin{pmatrix} 0 & 0 & \sigma^C_{xz} \\ 0 & 0 & \sigma^C_{yz} \\ \sigma^C_{zx} & \sigma^C_{zy} & 0 \end{pmatrix}$
$\mathcal{T}$-odd no soc	$\begin{pmatrix} \sigma^A_{xx} & \sigma^A_{yx} & 0 \\ \sigma^A_{yx} & \sigma^A_{xx} - \frac{2\sqrt{3}\sigma^A_{yx}}{3} & 0 \\ 0 & 0 & 0 \end{pmatrix}$	$\begin{pmatrix} -\frac{\sigma^A_{xx}}{2} + \frac{\sqrt{3}\sigma^A_{yx}}{2} & -\frac{\sqrt{3}\sigma^A_{xx}}{2} + \frac{3\sigma^A_{yx}}{2} & 0 \\ \frac{\sqrt{3}\sigma^A_{xx}}{2} + \frac{\sigma^A_{yx}}{2} & -\frac{\sigma^A_{xx}}{2} - \frac{\sqrt{3}\sigma^A_{yx}}{6} & 0 \\ 0 & 0 & 0 \end{pmatrix}$	$\begin{pmatrix} -\frac{\sigma^A_{xx}}{2} + \frac{\sqrt{3}\sigma^A_{yx}}{2} & -\frac{\sqrt{3}\sigma^A_{xx}}{2} + \frac{3\sigma^A_{yx}}{2} & 0 \\ \frac{\sqrt{3}\sigma^A_{xx}}{2} + \frac{\sigma^A_{yx}}{2} & -\frac{\sigma^A_{xx}}{2} - \frac{\sqrt{3}\sigma^A_{yx}}{6} & 0 \\ 0 & 0 & 0 \end{pmatrix}$
$\mathcal{T}$-odd soc	$\begin{pmatrix} \sigma^A_{xx} & \sigma^A_{yx} & 0 \\ \sigma^A_{yx} & \sigma^A_{xx} - \frac{2\sqrt{3}\sigma^A_{yx}}{3} & 0 \\ 0 & 0 & \sigma^A_{zz} \end{pmatrix}$	$\begin{pmatrix} \sigma^B_{xx} & \sigma^B_{xy} & 0 \\ \sigma^B_{yx} & \sigma^B_{yy} & 0 \\ 0 & 0 & \sigma^B_{zz} \end{pmatrix}$	$\begin{pmatrix} \sigma^C_{xx} & \sigma^C_{xy} & 0 \\ \sigma^C_{yx} & \sigma^C_{yy} & 0 \\ 0 & 0 & \sigma^C_{zz} \end{pmatrix}$

TABLE I. The symmetry tensors for Mn_3Sn. A, B, C are the different sublattices as denoted in Fig. 2 of the main text.

	A	B	C
$\mathcal{T}$-even no soc	$\begin{pmatrix} 0 & 0 & 0 \\ 0 & 0 & 0 \\ \sigma^A_{zx} & \sigma^A_{zy} & \sigma^A_{zz} \end{pmatrix}$	$\begin{pmatrix} 0 & 0 & 0 \\ 0 & 0 & 0 \\ \sigma^A_{zx} & -\sigma^A_{zy} & -\sigma^A_{zz} \end{pmatrix}$	$\begin{pmatrix} 0 & 0 & 0 \\ 0 & 0 & 0 \\ \sigma^C_{zx} & 0 & 0 \end{pmatrix}$
$\mathcal{T}$-even soc	$\begin{pmatrix} \sigma^A_{xx} & -\frac{2\sqrt{3}\sigma^A_{xx}}{3} - \sigma^A_{yx} & \sigma^A_{xz} \\ \sigma^A_{yx} & -\sigma^A_{xx} & -\sqrt{3}\sigma^A_{xz} \\ \sigma^A_{zx} & -\sqrt{3}\sigma^A_{zx} & 0 \end{pmatrix}$	$\begin{pmatrix} -\sigma^A_{xx} & -\frac{2\sqrt{3}\sigma^A_{xx}}{3} - \sigma^A_{yx} & \sigma^A_{xz} \\ \sigma^A_{yx} & \sigma^A_{xx} & \sqrt{3}\sigma^A_{xz} \\ \sigma^A_{zx} & \sqrt{3}\sigma^A_{zx} & 0 \end{pmatrix}$	$\begin{pmatrix} 0 & \sigma^C_{xy} & \sigma^C_{xz} \\ \sigma^C_{yx} & 0 & 0 \\ \sigma^C_{zx} & 0 & 0 \end{pmatrix}$
$\mathcal{T}$-odd no soc	$\begin{pmatrix} \sigma^A_{xx} & \sigma^A_{xy} & \sigma^A_{xz} \\ \sigma^A_{yx} & \sigma^A_{yy} & \sigma^A_{yz} \\ 0 & 0 & 0 \end{pmatrix}$	$\begin{pmatrix} \sigma^A_{xx} & -\sigma^A_{xy} & -\sigma^A_{xz} \\ -\sigma^A_{yx} & \sigma^A_{yy} & \sigma^A_{yz} \\ 0 & 0 & 0 \end{pmatrix}$	$\begin{pmatrix} \sigma^C_{xx} & 0 & 0 \\ 0 & \sigma^C_{yy} & \sigma^C_{yz} \\ 0 & 0 & 0 \end{pmatrix}$
$\mathcal{T}$-odd soc	$\begin{pmatrix} \sigma^A_{xx} & \sigma^A_{yx} & \sigma^A_{xz} \\ \sigma^A_{yx} & \sigma^A_{xx} - \frac{2\sqrt{3}\sigma^A_{yx}}{3} & \frac{\sqrt{3}\sigma^A_{xz}}{3} \\ \sigma^A_{zx} & \frac{\sqrt{3}\sigma^A_{zx}}{3} & \sigma^A_{zz} \end{pmatrix}$	$\begin{pmatrix} \sigma^A_{xx} & -\sigma^A_{yx} & -\sigma^A_{xz} \\ -\sigma^A_{yx} & \sigma^A_{xx} - \frac{2\sqrt{3}\sigma^A_{yx}}{3} & \frac{\sqrt{3}\sigma^A_{xz}}{3} \\ -\sigma^A_{zx} & \frac{\sqrt{3}\sigma^A_{zx}}{3} & \sigma^A_{zz} \end{pmatrix}$	$\begin{pmatrix} \sigma^C_{xx} & 0 & 0 \\ 0 & \sigma^C_{yy} & \sigma^C_{yz} \\ 0 & \sigma^C_{zy} & \sigma^C_{zz} \end{pmatrix}$

TABLE II. Symmetry tensors for the Mn_3Ir bilayer.

sublattice	no SOC	SOC
A	$\begin{pmatrix} 0 & 0 & 0 \\ 0 & 0 & 0 \\ 0 & \sigma^A_{zy} & 0 \end{pmatrix}$	$\begin{pmatrix} 0 & \sigma^A_{xy} & 0 \\ \sigma^A_{yx} & 0 & \sigma^A_{yz} \\ 0 & \sigma^A_{zy} & 0 \end{pmatrix}$
B	$\begin{pmatrix} 0 & 0 & 0 \\ 0 & 0 & 0 \\ -\frac{\sqrt{3}\sigma^A_{zy}}{2} & -\frac{\sigma^A_{zy}}{2} & 0 \end{pmatrix}$	$\begin{pmatrix} \frac{\sqrt{3}\sigma^A_{xy}}{4} + \frac{\sqrt{3}\sigma^A_{yx}}{4} & \frac{\sigma^A_{xy}}{4} - \frac{3\sigma^A_{yx}}{4} & -\frac{\sqrt{3}\sigma^A_{yz}}{2} \\ -\frac{3\sigma^A_{xy}}{4} + \frac{\sigma^A_{yx}}{4} & -\frac{\sqrt{3}\sigma^A_{xy}}{4} - \frac{\sqrt{3}\sigma^A_{yx}}{4} & -\frac{\sigma^A_{yz}}{2} \\ -\frac{\sqrt{3}\sigma^A_{zy}}{2} & -\frac{\sigma^A_{zy}}{2} & 0 \end{pmatrix}$
C	$\begin{pmatrix} 0 & 0 & 0 \\ 0 & 0 & 0 \\ \frac{\sqrt{3}\sigma^A_{zy}}{2} & -\frac{\sigma^A_{zy}}{2} & 0 \end{pmatrix}$	$\begin{pmatrix} -\frac{\sqrt{3}\sigma^A_{xy}}{4} - \frac{\sqrt{3}\sigma^A_{yx}}{4} & \frac{\sigma^A_{xy}}{4} - \frac{3\sigma^A_{yx}}{4} & \frac{\sqrt{3}\sigma^A_{yz}}{2} \\ -\frac{3\sigma^A_{xy}}{4} + \frac{\sigma^A_{yx}}{4} & \frac{\sqrt{3}\sigma^A_{xy}}{4} + \frac{\sqrt{3}\sigma^A_{yx}}{4} & -\frac{\sigma^A_{yz}}{2} \\ \frac{\sqrt{3}\sigma^A_{zy}}{2} & -\frac{\sigma^A_{zy}}{2} & 0 \end{pmatrix}$
D	$\begin{pmatrix} 0 & 0 & 0 \\ 0 & 0 & 0 \\ 0 & -\sigma^A_{zy} & 0 \end{pmatrix}$	$\begin{pmatrix} 0 & \sigma^A_{xy} & 0 \\ \sigma^A_{yx} & 0 & -\sigma^A_{yz} \\ 0 & -\sigma^A_{zy} & 0 \end{pmatrix}$
E	$\begin{pmatrix} 0 & 0 & 0 \\ 0 & 0 & 0 \\ \frac{\sqrt{3}\sigma^A_{zy}}{2} & \frac{\sigma^A_{zy}}{2} & 0 \end{pmatrix}$	$\begin{pmatrix} \frac{\sqrt{3}\sigma^A_{xy}}{4} + \frac{\sqrt{3}\sigma^A_{yx}}{4} & \frac{\sigma^A_{xy}}{4} - \frac{3\sigma^A_{yx}}{4} & \frac{\sqrt{3}\sigma^A_{yz}}{2} \\ -\frac{3\sigma^A_{xy}}{4} + \frac{\sigma^A_{yx}}{4} & -\frac{\sqrt{3}\sigma^A_{xy}}{4} - \frac{\sqrt{3}\sigma^A_{yx}}{4} & \frac{\sigma^A_{yz}}{2} \\ \frac{\sqrt{3}\sigma^A_{zy}}{2} & \frac{\sigma^A_{zy}}{2} & 0 \end{pmatrix}$
F	$\begin{pmatrix} 0 & 0 & 0 \\ 0 & 0 & 0 \\ -\frac{\sqrt{3}\sigma^A_{zy}}{2} & \frac{\sigma^A_{zy}}{2} & 0 \end{pmatrix}$	$\begin{pmatrix} -\frac{\sqrt{3}\sigma^A_{xy}}{4} - \frac{\sqrt{3}\sigma^A_{yx}}{4} & \frac{\sigma^A_{xy}}{4} - \frac{3\sigma^A_{yx}}{4} & -\frac{\sqrt{3}\sigma^A_{yz}}{2} \\ -\frac{3\sigma^A_{xy}}{4} + \frac{\sigma^A_{yx}}{4} & \frac{\sqrt{3}\sigma^A_{xy}}{4} + \frac{\sqrt{3}\sigma^A_{yx}}{4} & \frac{\sigma^A_{yz}}{2} \\ -\frac{\sqrt{3}\sigma^A_{zy}}{2} & \frac{\sigma^A_{zy}}{2} & 0 \end{pmatrix}$

TABLE III. The $\mathcal{T}$-even Edelstein effect symmetry tensors for the $LuFeO_3$.

sublattice	no SOC	SOC
A	$\begin{pmatrix} \sigma^A_{xx} & 0 & \sigma^A_{xz} \\ 0 & \sigma^A_{yy} & 0 \\ 0 & 0 & 0 \end{pmatrix}$	$\begin{pmatrix} \sigma^A_{xx} & 0 & \sigma^A_{xz} \\ 0 & \sigma^A_{yy} & 0 \\ \sigma^A_{zx} & 0 & \sigma^A_{zz} \end{pmatrix}$
B	$\begin{pmatrix} \frac{\sigma^A_{xx}}{4} + \frac{3\sigma^A_{yy}}{4} & -\frac{\sqrt{3}\sigma^A_{xx}}{4} + \frac{\sqrt{3}\sigma^A_{yy}}{4} & -\frac{\sigma^A_{xz}}{2} \\ -\frac{\sqrt{3}\sigma^A_{xx}}{4} + \frac{\sqrt{3}\sigma^A_{yy}}{4} & \frac{3\sigma^A_{xx}}{4} + \frac{\sigma^A_{yy}}{4} & \frac{\sqrt{3}\sigma^A_{xz}}{2} \\ 0 & 0 & 0 \end{pmatrix}$	$\begin{pmatrix} \frac{\sigma^A_{xx}}{4} + \frac{3\sigma^A_{yy}}{4} & -\frac{\sqrt{3}\sigma^A_{xx}}{4} + \frac{\sqrt{3}\sigma^A_{yy}}{4} & -\frac{\sigma^A_{xz}}{2} \\ -\frac{\sqrt{3}\sigma^A_{xx}}{4} + \frac{\sqrt{3}\sigma^A_{yy}}{4} & \frac{3\sigma^A_{xx}}{4} + \frac{\sigma^A_{yy}}{4} & \frac{\sqrt{3}\sigma^A_{xz}}{2} \\ -\frac{\sigma^A_{zx}}{2} & \frac{\sqrt{3}\sigma^A_{zx}}{2} & \sigma^A_{zz} \end{pmatrix}$
C	$\begin{pmatrix} \frac{\sigma^A_{xx}}{4} + \frac{3\sigma^A_{yy}}{4} & \frac{\sqrt{3}\sigma^A_{xx}}{4} - \frac{\sqrt{3}\sigma^A_{yy}}{4} & -\frac{\sigma^A_{xz}}{2} \\ \frac{\sqrt{3}\sigma^A_{xx}}{4} - \frac{\sqrt{3}\sigma^A_{yy}}{4} & \frac{3\sigma^A_{xx}}{4} + \frac{\sigma^A_{yy}}{4} & -\frac{\sqrt{3}\sigma^A_{xz}}{2} \\ 0 & 0 & 0 \end{pmatrix}$	$\begin{pmatrix} \frac{\sigma^A_{xx}}{4} + \frac{3\sigma^A_{yy}}{4} & \frac{\sqrt{3}\sigma^A_{xx}}{4} - \frac{\sqrt{3}\sigma^A_{yy}}{4} & -\frac{\sigma^A_{xz}}{2} \\ \frac{\sqrt{3}\sigma^A_{xx}}{4} - \frac{\sqrt{3}\sigma^A_{yy}}{4} & \frac{3\sigma^A_{xx}}{4} + \frac{\sigma^A_{yy}}{4} & -\frac{\sqrt{3}\sigma^A_{xz}}{2} \\ -\frac{\sigma^A_{zx}}{2} & -\frac{\sqrt{3}\sigma^A_{zx}}{2} & \sigma^A_{zz} \end{pmatrix}$
D	$\begin{pmatrix} \sigma^A_{xx} & 0 & -\sigma^A_{xz} \\ 0 & \sigma^A_{yy} & 0 \\ 0 & 0 & 0 \end{pmatrix}$	$\begin{pmatrix} \sigma^A_{xx} & 0 & -\sigma^A_{xz} \\ 0 & \sigma^A_{yy} & 0 \\ -\sigma^A_{zx} & 0 & \sigma^A_{zz} \end{pmatrix}$
E	$\begin{pmatrix} \frac{\sigma^A_{xx}}{4} + \frac{3\sigma^A_{yy}}{4} & -\frac{\sqrt{3}\sigma^A_{xx}}{4} + \frac{\sqrt{3}\sigma^A_{yy}}{4} & \frac{\sigma^A_{xz}}{2} \\ -\frac{\sqrt{3}\sigma^A_{xx}}{4} + \frac{\sqrt{3}\sigma^A_{yy}}{4} & \frac{3\sigma^A_{xx}}{4} + \frac{\sigma^A_{yy}}{4} & -\frac{\sqrt{3}\sigma^A_{xz}}{2} \\ 0 & 0 & 0 \end{pmatrix}$	$\begin{pmatrix} \frac{\sigma^A_{xx}}{4} + \frac{3\sigma^A_{yy}}{4} & -\frac{\sqrt{3}\sigma^A_{xx}}{4} + \frac{\sqrt{3}\sigma^A_{yy}}{4} & \frac{\sigma^A_{xz}}{2} \\ -\frac{\sqrt{3}\sigma^A_{xx}}{4} + \frac{\sqrt{3}\sigma^A_{yy}}{4} & \frac{3\sigma^A_{xx}}{4} + \frac{\sigma^A_{yy}}{4} & -\frac{\sqrt{3}\sigma^A_{xz}}{2} \\ \frac{\sigma^A_{zx}}{2} & -\frac{\sqrt{3}\sigma^A_{zx}}{2} & \sigma^A_{zz} \end{pmatrix}$
F	$\begin{pmatrix} \frac{\sigma^A_{xx}}{4} + \frac{3\sigma^A_{yy}}{4} & \frac{\sqrt{3}\sigma^A_{xx}}{4} - \frac{\sqrt{3}\sigma^A_{yy}}{4} & \frac{\sigma^A_{xz}}{2} \\ \frac{\sqrt{3}\sigma^A_{xx}}{4} - \frac{\sqrt{3}\sigma^A_{yy}}{4} & \frac{3\sigma^A_{xx}}{4} + \frac{\sigma^A_{yy}}{4} & \frac{\sqrt{3}\sigma^A_{xz}}{2} \\ 0 & 0 & 0 \end{pmatrix}$	$\begin{pmatrix} \frac{\sigma^A_{xx}}{4} + \frac{3\sigma^A_{yy}}{4} & \frac{\sqrt{3}\sigma^A_{xx}}{4} - \frac{\sqrt{3}\sigma^A_{yy}}{4} & \frac{\sigma^A_{xz}}{2} \\ \frac{\sqrt{3}\sigma^A_{xx}}{4} - \frac{\sqrt{3}\sigma^A_{yy}}{4} & \frac{3\sigma^A_{xx}}{4} + \frac{\sigma^A_{yy}}{4} & \frac{\sqrt{3}\sigma^A_{xz}}{2} \\ \frac{\sigma^A_{zx}}{2} & \frac{\sqrt{3}\sigma^A_{zx}}{2} & \sigma^A_{zz} \end{pmatrix}$

TABLE IV. The $\mathcal{T}$-odd Edelstein effect symmetry tensors for the $LuFeO_3$.

Mn_3Sn CALCULATIONS

In Fig. S1 we give the calculation of the local Edelstein effect in Mn_3Sn for the sublattices A, B and C. The sublattices A', B' and C' are connected by inversion symmetry to the sublattices shown here and thus they must have the opposite Edelstein effect.

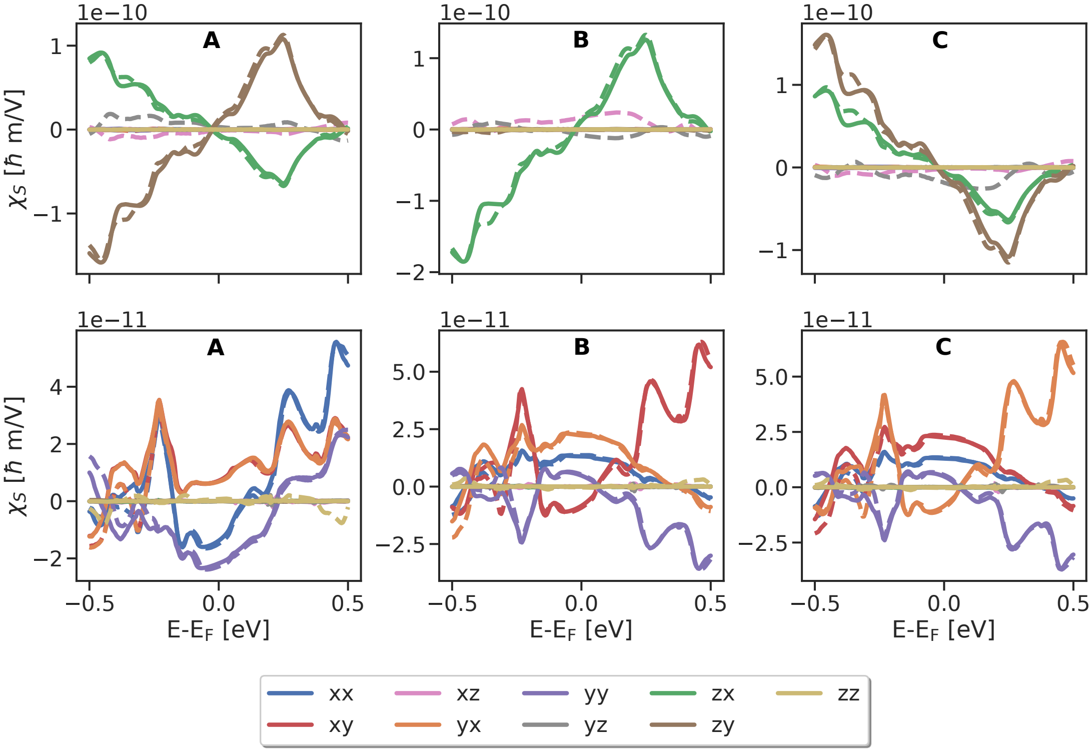

FIG. S1. Calculation of the local Edelstein effect in Mn_3Sn with (dashed lines) and without (solid lines) spin-orbit coupling. The labeling of the sublattices is given in Fig. 2 of the main text.

LUFEO$_3$ CALCULATIONS

Here we give the result of the Edelstein effect calculations in $LuFeO_3$ for all the sublattices. In Fig. S2 we label the different sublattices and in Fig. S3 we show the result of the calculations.

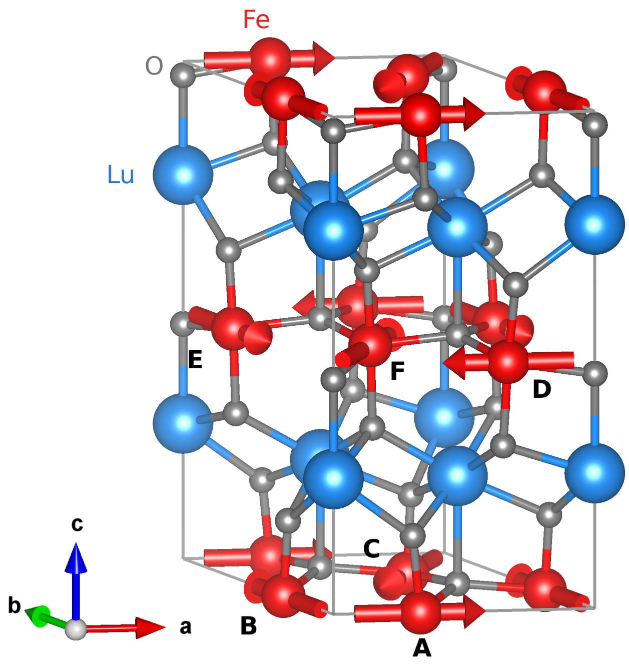

FIG. S2. $LuFeO_3$ structure with sublattice labels.

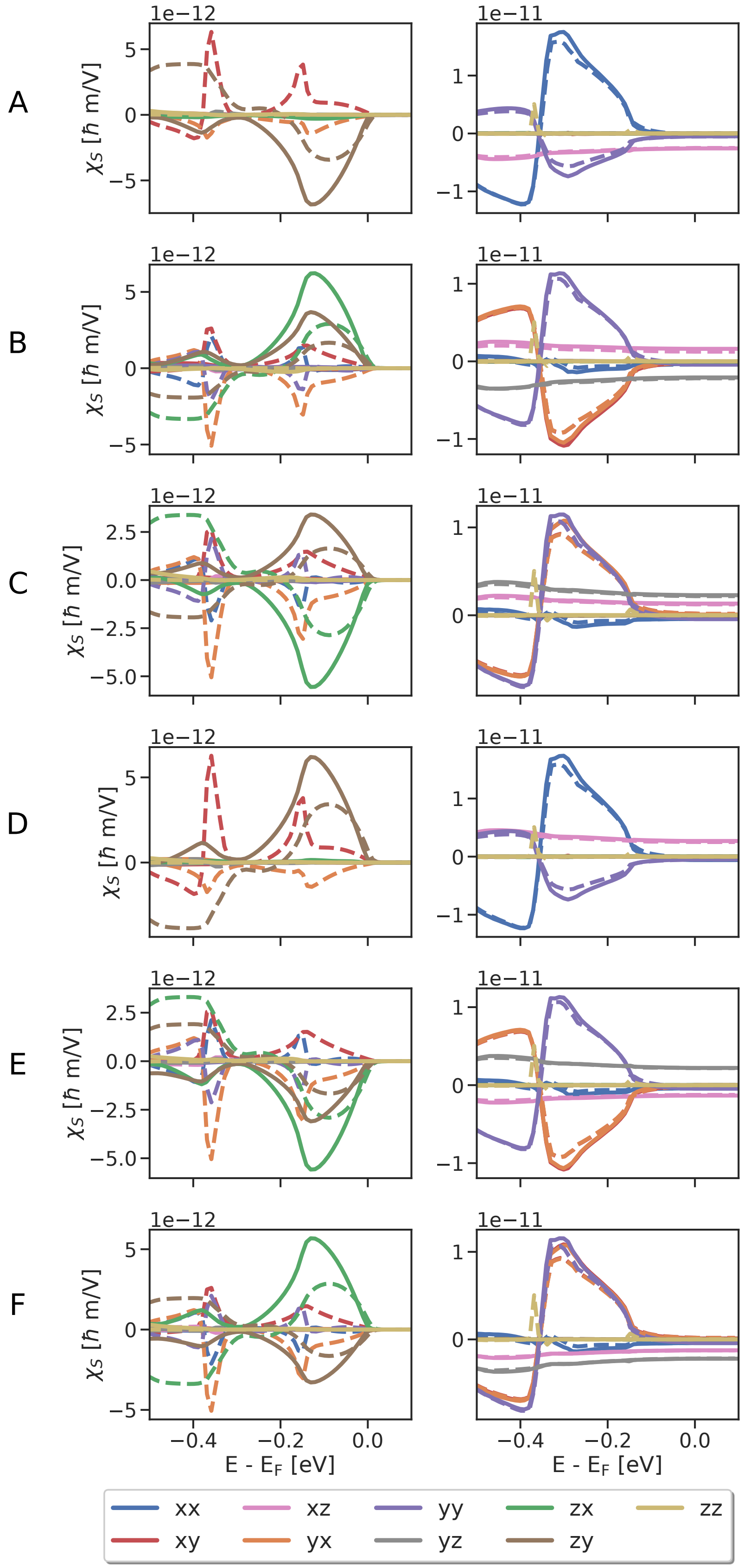

FIG. S3. Edelstein effect calculations in $LuFeO_3$ with and without spin-orbit coupling. The left/right column corresponds to the $\mathcal{T}$-even and $\mathcal{T}$-odd components respectively.

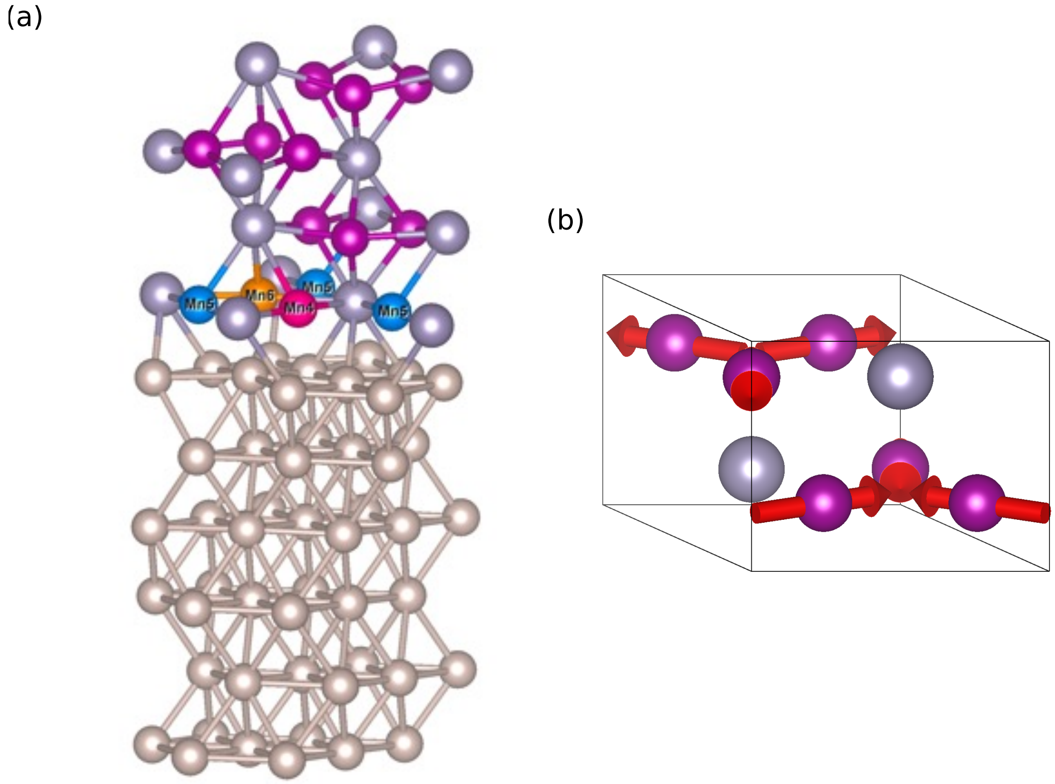

FIG. S4. (a) Mn_3Sn/Ru(0001) structure with sublattice labels. (b) The magnetic order used for the slab calculations.

MN_3SN/RU(0001) DFT CALCULATIONS

For illustration, we include here also preliminary results of Edelstein effect calculations on Mn_3Sn/Ru(0001) slab. We note that these calculations use a slightly different magnetic phase for Mn_3Sn, as shown in Fig. S4(b). The present calculations are performed in the framework of the density functional theory (DFT) using VASP code. The parameter conditions mirror those outlined in the main text for bulk calculations of Mn_3Sn and $LuFeO_3$. The Brillouin zone integration is executed employing an ($11\times11\times1$) k-points mesh. The interface model was calculated using a Mn_3Sn/Ru surface, which represents a slab with 4 Mn_3Sn and 6 Ru layers, stacked in the hexagonal growth direction [0001]. The slabs are separated from their periodic replicas in the [0001] direction by a 17 Åvacuum layer. During the geometry optimization stage, relaxation of all slab atoms is performed until the forces acting on them do not exceed 0.01 eV/Å. All calculations were carried out without considering the spin-orbit coupling interaction. In the Wannierization process, we employed the d orbitals for the Ru and Mn atoms, while the p orbitals were utilized for the Sn atoms. The frozen energy window was set to $E_F = +1eV$. In our linear response calculations, we employed the Linres code with a $480\times480\times1$ k-mesh.

The results for the $\mathcal{T}$-odd and $\mathcal{T}$-even components of the torque, projected on the three magnetic sublattices as indicated in the sketch of the heterostructure, Fig. S4, are reported in Figs. S5 and S6, respectively. Two comments are in order: first, the overall magnitude of the effect is comparable to that reported in Fig. 5 of the main text and computed with a model system. From our viewpoint, this is not surprising as the band structure of this metallic heterostructure is very dense and the spin transport is rather governed by the interplay between the magnetic configuration, the bandwidth, and the interfacial orbital hybridization. In the presence of a large number of orbitals, as is the case here, one expects that the details in the band structure should have a reduced impact.

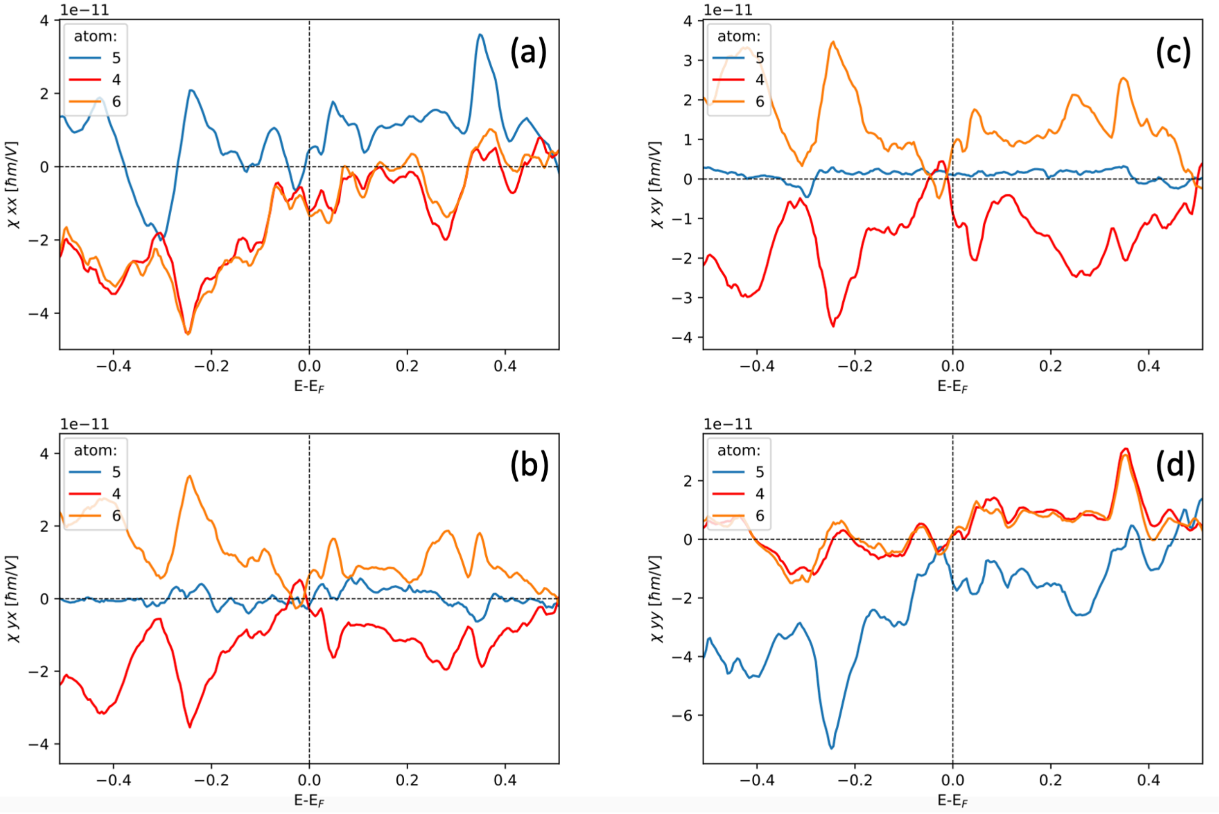

FIG. S5. $\mathcal{T}$-odd components of the local Edelstein effect projected on the three magnetic sublattices, as indicated on the crystal structure.

MN$_3$SN/RU(0001) TIGHT-BINDING CALCULATIONS

Since the DFT calculations of the bilayer slab systems are numerically very demanding we have also performed tight-binding calculations of the Mn_3Sn/Ru bilayer system. This allows considering larger systems than in the DFT calculations. These calculations allow comparing with the DFT calculations, although we note that the setup of the system is not exactly the same and the DFT calculations use a slightly different magnetic structure than considered here and for the bulk calculations. The calculations utilize a simple non-relativistic tight-binding model similar to the one used for the 3Q system and the Mn_3Ir bilayer calculations defined in Eq. (3) of the main text. The only distinction is that this system require hoppings beyond nearest neighbor. We consider up to 5-th nearest neighbor and scale the hoppings such that $t = 1\,\text{eV} * d_{\text{nn}}^2/d$, where d_{nn} is the nearest neighbour distance and d is the distance of the given hopping. We consider $J = 1.7\,\text{eV}$ as for the Mn_3Ir bilayer and we set $E_F = 0.5\,\text{eV}$. For these calculations we do not include the Sn atoms as they don't change the symmetry of the structure and are not necessary for the simple model we use. Apart from the omission of the Sn atoms the structure is analogous to the one used for the DFT calculations S4, except with different number of layers. We use 3 atomic layers for Ru and 10 unit cells (corresponding to 20 Mn layers) of Mn_3Sn.

The results of the calculations are shown in Fig. S7. These show that as expected the interfaces break the global inversion symmetry, which means that the Edelstein effect within each unit cell does not cancel out. The symmetry breaking is largest close to the interfaces. Such Edelstein effect will likely lead to a torque that can efficiently manipulate tha magnetic order, however, studying the magnetic dynamics induced by the torque is beyond the scope of this work.

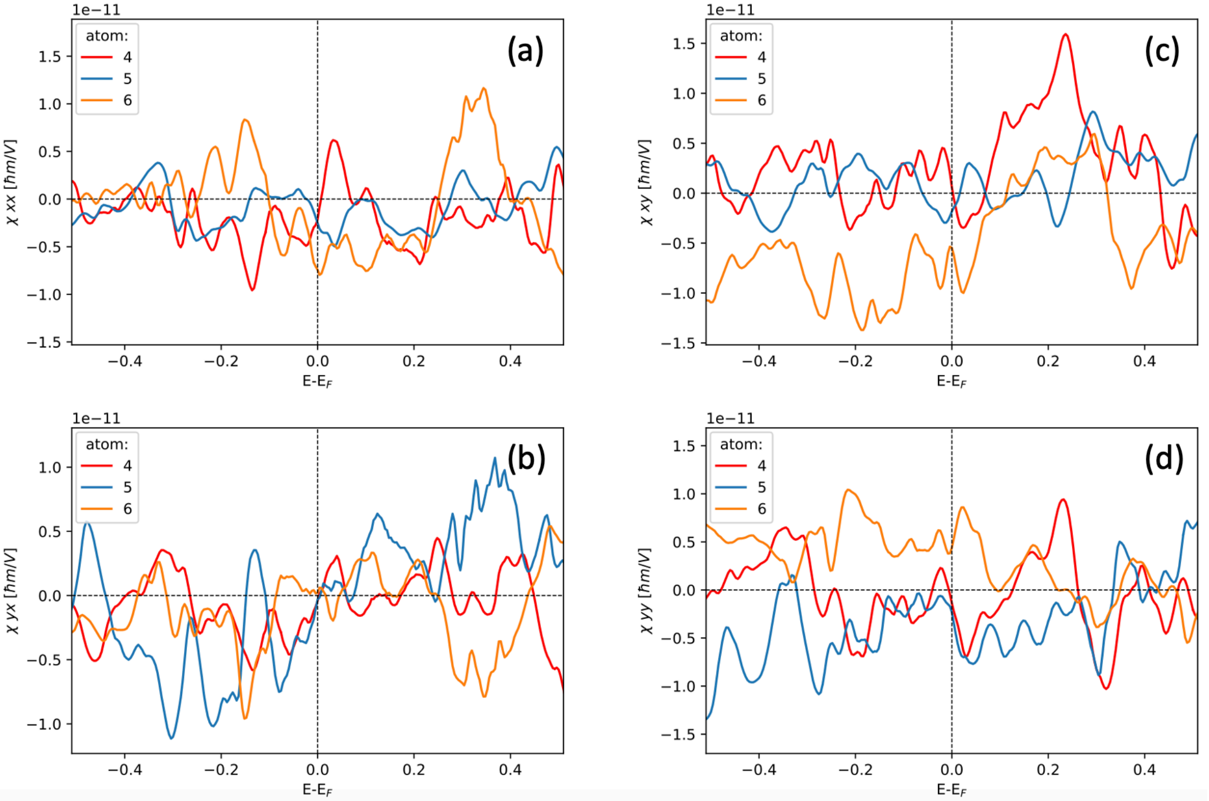

FIG. S6. $\mathcal{T}$-even components of the local Edelstein effect projected on the three magnetic sublattices, as indicated on the crystal structure.

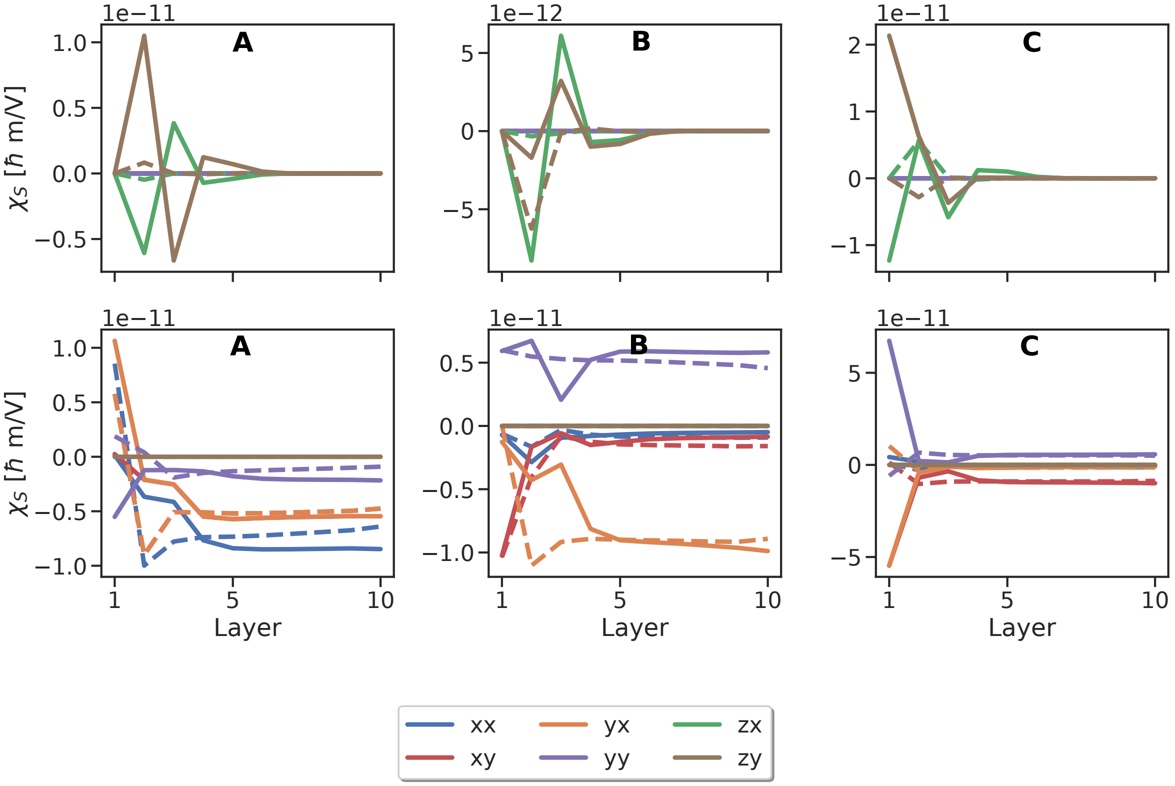

FIG. S7. The results of the Edelstein effect in the tight-binding model Mn_3Sn/Ru calculations. The top row shows the $\mathcal{T}$-even calculations and the bottom row the $\mathcal{T}$-odd calculations. The dashed line shows the comparison to the sublattices that would be connected by inversion symmetry in the bulk: for example, for the sublattice A, the solid line shows the result for sublattice A, whereas the dashed line shows *minus* the result of the sublattice A'. Thus if the solid and dashed lines are on top of each other this shows that the Edelstein effect satisfies the bulk inversion symemtry, when they differ it shows the inversion symmetry breaking.

NON-RELATIVISTIC SYMMETRY ANALYSIS

For the non-relativistic symmetry analysis we use the Symmetr code [1] in which we have implemented an alghorithm for identifying the non-relativistic symmetry operations. Once the non-relativistic symmetry operations are identified the procedure for obtaining the symmetry restricted form of the response tensors is the same as in the case of relativistic symmetry, as described in Ref. [2].

In the absence of spin-orbit coupling, the symmetry is described by the so-called spin-groups [3, 4]. These differ from the magnetic space groups that describe the relativistic symmetry in that the spin and spatial rotations are decoupled. That is, with spin-orbit coupling a rotation is composed of a real space rotation and equivalent spin rotation. Without spin-orbit coupling, however, the spin rotation can be different from the real space part. Here we briefly describe the procedure for obtaining all the non-relativistic symmetries. A detailed description of the algorithm and the code will be published elsewhere.

In the absence of spin-orbit coupling, the symmetry operations for a lattice are composed of rotations (2-fold, 3-fold, 4-fold and 6-fold), spatial inversion, pure spin rotation (that can be in general arbitrary) and time-reversal. In a non-magnetic system, the spin group is a product of a crystallographic space group, the group containing all spin rotations and time-reversal. In a magnetic system, the symmetry is reduced. For example, magnetic systems are not invariant under time-reversal or an arbitrary spin-rotation.

Since all the symmetry operations of the magnetic system must also be symmetry operations of the non-magnetic system, we use the symmetry operations of the non-magnetic system as a starting point. We assume that the symmetry of the system is described by assigning magnetic moments to each site. This is usually satisfied; however, it may also happen that the whole magnetization density needs to be taken into account. In such a case, the symmetry analysis based on the approach used here would be higher than in the real system. However, the general principles outlined here would still apply.

The procedure for obtaining the non-relativistic symmetry operations is as follows:

1. Identify all the non-magnetic symmetry operations. For this we use the Findsym code. We consider only the spatial part of the symmetry operations and we need to find any spin rotations that, together with the spatial component, are symmetries of the magnetic system.

2. Because the spatial and spin transformations are decoupled, the only aspect of the spatial symmetry operations that matters for the spin rotation is how they permute the magnetic atoms. That is, under a combined spin-rotation R_s and spatial transformation R, the magnetic moments of the system will transform as $\mathbf{M}_i \to R_s \mathbf{M}_{p_i}$, where p_i denotes the permutation corresponding to R. The combined operation will be a symmetry of the system if for all moments $\mathbf{M}_i \to R_s \mathbf{M}_{p_i}$.

   For example, a symmetry operation may transform atom A to atom B, atom B to atom C and atom C to atom A. We denote this operation as $A \to B \to C \to A$ and refer to as a permutation chain. Each permutation chain must end with the same atom as the one it starts with. Each symmetry operation is described by one or more such chains.

3. Now for every permutation chain we have to find the spin-rotations that leave it invariant, that is spin-rotations such that $R_s \mathbf{M}_{c_i} = \mathbf{M}_{c_{i+1}}$, where $\mathbf{M}_{c_i}$ denotes the magnetic moment of each chain. This has to be done separately with and without time-reversal. In the following we assume that all the moments have same magnitude, since otherwise they cannot be symmetry related.

   Since the last element of the chain must be the same as the first, we have a condition:

$$\text{Without time-reversal:} \quad \mathbf{M}_A = R_s^n \mathbf{M}_A \tag{1}$$

$$\text{With time-reversal:} \quad \mathbf{M}_A = (-1)^n R_s^n \mathbf{M}_A \tag{2}$$

   where n is the number of unique atoms in the chain (that is for chain $A \to B \to C \to A$, the length is 3). From this, it is possible to determine the necessary conditions for the existence of a spin-rotation that leaves the chain invariant.

   If the chain is collinear then the symmetry analysis is quite simple. The only two relevant symmetries are an arbitrary rotation around the collinear axis $R_s^{||}$ and a π rotation around any perpendicular axis $R_s^{\perp}$. The system can either be invariant under $R_s^{||}$ or under $R_s^{\perp} R_s^{||}$. In this case, the chain has a continuous spin-rotation symmetry.

   For a non-collinear system, the condition for the rotation angle θ are:

- Without time-reversal or with time-reversal and n even: $\theta = \frac{i\pi}{n}$, where i is an even integer.
- With time-reversal and n odd: $\theta = \frac{i\pi}{n}$, where i an odd integer.

4. For each permutation pair in the chain, we now have to determine the spin-rotations that leave it invariant taking into account the restriction on θ. We do this by first considering each pair separately. That is, for example, for the chain $A \to B \to C \to A$ we have to find spin-rotations R_s that satisfy $R_sA = B$, $R_sB = C$ and $R_sC = A$ and then find the spin-rotations that are common to all pairs.

   In general, it can be shown that a rotation R_s with an angle θ connecting two vectors $\mathbf{M}_A$ and $\mathbf{M}_B$, will exist if $\mathbf{M}_A \cdot \mathbf{M}_B \geq \cos(\theta)$, that is the angle between the two vectors must greater or equal to θ. The rotation axes will be given by:

$$
\begin{aligned}
\mathbf{n}^{1+} &= \cos(\alpha)\frac{\mathbf{M}_A + \mathbf{M}_B}{||\mathbf{M}_A + \mathbf{M}_B||} + \sin(\alpha)\frac{\mathbf{M}_A \times \mathbf{M}_B}{||\mathbf{M}_A \times \mathbf{M}_B||}, \\
\mathbf{n}^{1-} &= -\cos(\alpha)\frac{\mathbf{M}_A + \mathbf{M}_B}{||\mathbf{M}_A + \mathbf{M}_B||} - \sin(\alpha)\frac{\mathbf{M}_A \times \mathbf{M}_B}{||\mathbf{M}_A \times \mathbf{M}_B||}. \\
\mathbf{n}^{2+} &= \cos(\alpha)\frac{\mathbf{M}_A + \mathbf{M}_B}{||\mathbf{M}_A + \mathbf{M}_B||} - \sin(\alpha)\frac{\mathbf{M}_A \times \mathbf{M}_B}{||\mathbf{M}_A \times \mathbf{M}_B||}, \\
\mathbf{n}^{2-} &= -\cos(\alpha)\frac{\mathbf{M}_A + \mathbf{M}_B}{||\mathbf{M}_A + \mathbf{M}_B||} + \sin(\alpha)\frac{\mathbf{M}_A \times \mathbf{M}_B}{||\mathbf{M}_A \times \mathbf{M}_B||},
\end{aligned}
\tag{3}
$$

   where

$$
\alpha = \arccos\left(\sqrt{\frac{2\left(\mathbf{M}_A \cdot \mathbf{M}_B - \cos(\theta)\right)}{(1-\cos(\theta))(1+\mathbf{M}_A \cdot \mathbf{M}_B)}}\right). \tag{4}
$$

   The $\pm$ axes correspond to opposite sense of rotation. In general only one of the two is correct. It can easily be checked, which one is the correct one for a given $\mathbf{M}_A$, $\mathbf{M}_B$ and θ.

5. Once we have the spin-rotations that are symmetries of each permutation chain, we find the spin-rotations (if any) that are common to all chains. These, together with the spatial part of the symmetry corresponding the permutation and potentially time-reversal, are symmetries of the system.

SYMMETRIZATION OF TENSORS

Here we briefly describe the procedure we use for obtaining the symmetry restriced form of response tensors. This has also been discussed in Ref. [2]. The basic process is:

1. Obtain the list of symmetry operations for a given crystal and magnetic structure. For this we use the alghorithm described in the previous section.

2. For each symmetry operation we determine the transformation of the reponse tensor and set up a system of linear equations that have to be satisfied for the tensor. For symmetry operations containing time-reversal we have to separate the tensor into $\mathcal{T}$-even and $\mathcal{T}$-odd parts. In general for the Edelstein effect response tensor, for symmetry operation without time-reversal we can write the transformation as:

$$
\chi_{ij} = R^s_{ik}\chi_{kl}(R^E)^{-1}_{lj}, \tag{5}
$$

   where R^s, R^E are the matrices representing the transformation of the spin and the electric field under the symmetry operation. For symmetry operations with time-reversal the transformation can be written as:

$$
\chi^{\text{even}}_{ij} = -R^s_{ik}\chi^{\text{even}}_{kl}(R^E)^{-1}_{lj}, \tag{6}
$$

$$
\chi^{\text{odd}}_{ij} = R^s_{ik}\chi^{\text{odd}}_{kl}(R^E)^{-1}_{lj}, \tag{7}
$$

   We note that since spin is odd under time-reversal and the electric field is even, the $+$ sign corresponds to the odd part of the response tensor and $-$ to the even part.

3. Eqs. (5), (6),(7) form a set of linear equations for components of the response tensor, which have to be solved. This system of equations either has no solutions, in which case the response tensor is null or it has infinitely many solutions. These solutions can be parametrized with free variables and these free variables are then the independent components of the response tensor. The number of free variables is the dimension of the vector space formed by the solutions of the equation system.

   The system of equations that has to be solved can be written as:

$$Y\chi^v = 0, \tag{8}$$

   where χ^v is the vector formed from the components of the χ_{ij} matrix and Y is a matrix reprensenting the tranformation of the tensor by the symmetry operation. In the present case, where χ is a rank 2 tensor, Y is a 9x9 matrix. The easiest way how to solve this is using the Gaussian elimination, i.e. converting the matrix Y to reduced row echelon form. This allows directly eliminating the dependent tensor components. However, the Gaussian elimination is not numerically stable and thus for numerical solution it is better to use the SVD decomposition. We first use SVD decomposition to determine the null space of matrix Y, i.e. the vector space of all the solutions and then use the gaussian elimination on the basis vectors of the null space, which allows eliminating the dependent variables.

4. This procedure is then repeated for every symmetry operation in the group. It is sufficient to consider only the generators of the group since they fully determine the symmetry of the tensor.

This whole procedure is implemented in the open-source code Symmetr [1]. We describe in the next section how this code can be directly used for determining the symmetry of the materials from MAGNDATA.

SYMMETRY ANALYSIS FOR MATERIALS FROM MAGNDATA

We have used the approach outlined in the previous section to analyze the symmetry of the non-relativistic Edelstein effect for all the materials from MAGNDATA [5, 6]. In Table V we give only the information on whether each material has a net or a sublattice non-relativistic Edelstein effect. In a separate file 'sot_database_out.json' we give the full tensors for each material. This file can be loaded into python as follows:

```
import pandas as pd
df = pd.read_json('sot_database_out.json')
```

The column 'X_noso' contains the symmetry tensors. These can be converted into python dictionary using the 'eval' command. The individual entries of the dictionary are the even and odd symmetry tensors for the total and sublattice Edelstein effect. Note that we consider here only non-equivalent magnetic sites for the symmetry analysis.

The symmetry tensors can be converted into internal format used by the Symmetr code, which allows for easy printing or manipulation of the tensors. To do so the Symmetr python package must be installed, which can be done using pip. On linux:

```
pip install symmetr
```

For example, to convert, the total even tensor for the first row of the table:

```
from symmetr.tensors import Tensor
X = Tensor.load(eval(df.loc[0]['X_noso'])['tot']['even'])
X.pprint()
```

Alternatively the tensors for the MAGNDATA materials can be directly obtained using the Symmetr code by specifying their id. For example to obtain the non-relativistic symmetry of the Edelstein effect for $LuFeO_3$:

```
symmetr res s E --magndata 0.117 --noso
```

TABLE V: Non-relativistic Edelstein effect symmetry for materials from MAGNDATA. The index refers to the MAGNDATA index. For each material we show whether it has global inversion symmetry, whether it is antiferromagnetic, whether a global non-relativistic Edelstein effect is allowed or if it is allowed on some magnetic sublattice.

index	material	inversion	antiferromagnetic	total	sublattice
0.2	Cd2Os2O7	✓	✓	✗	✗
0.6	YMnO3	✗	✓	✓	✓
0.7	ScMnO3	✗	✓	✓	✓
0.8	ScMnO3	✗	✓	✓	✓
0.9	GdB4	✗	✓	✓	✓
0.10	DyFeO3	✗	✓	✓	✓
0.11	DyFeO3	✗	✗	✓	✓
0.12	U3(Al3Ru)4	✗	✓	✓	✓
0.17	FePO4	✗	✓	✓	✓
0.20	MnTe2	✓	✓	✗	✗
0.26	TmAgGe	✗	✗	✓	✓
0.27	Y(Fe2Ge)2	✗	✓	✓	✓
0.28	LiFe(SiO3)2	✗	✓	✓	✓
0.29	Er2Ti2O7	✓	✓	✗	✗
0.30	YbMnO3	✗	✓	✗	✓
0.31	HoMnO3	✗	✓	✓	✓
0.32	HoMnO3	✗	✓	✓	✓
0.33	HoMnO3	✗	✓	✓	✓
0.36	NiF2	✓	✗	✗	✗
0.37	U3Al2Si3	✗	✗	✓	✓
0.39	NaNd2RuO6	✓	✗	✗	✓
0.40	Mn2O3	✓	✓	✗	✓
0.41	Mn2O3	✓	✓	✗	✓
0.42	HoMnO3	✗	✓	✓	✓
0.43	HoMnO3	✗	✓	✓	✓
0.44	YMnO3	✗	✓	✗	✓
0.46	BaCaCo4O7	✗	✗	✓	✓
0.47	Gd2Sn2O7	✓	✓	✗	✗
0.48	Tb2Sn2O7	✓	✗	✗	✗
0.49	Ho2Ru2O7	✓	✗	✗	✗
0.51	Ho2Ru2O7	✓	✗	✗	✗
0.64	MnV2O4	✓	✗	✗	✓
0.69	Co2H25C15O7	✓	✗	✗	✓
0.70	Na3CoC2ClO6	✓	✓	✗	✗
0.74	Mn3CuN	✓	✓	✗	✗
0.77	Tb2Ti2O7	✓	✗	✗	✗
0.78	Ni(NO3)2	✓	✗	✗	✗
0.80	U2InPd2	✗	✓	✓	✓
0.81	U2SnPd2	✗	✓	✓	✓
0.85	KCo4(PO4)3	✓	✗	✗	✓
0.88	LiNiPO4	✗	✓	✓	✓
0.90	Rb2Fe2As2O9	✓	✓	✗	✓
0.91	Rb2Fe2As2O9	✓	✗	✗	✓
0.96	CoSO4	✓	✓	✗	✗
0.97	Fe(SbO2)2	✗	✓	✗	✗
0.101	Mn2GeO4	✓	✓	✗	✓
0.102	Mn2GeO4	✓	✓	✗	✓
0.103	Mn2GeO4	✓	✓	✗	✓
0.104	ErVO3	✓	✓	✗	✗
0.106	DyVO3	✓	✗	✗	✓
0.107	Ho2Ge2O7	✗	✓	✓	✓
0.108	Mn3Ir	✓	✓	✗	✗
0.109	Mn3Pt	✓	✓	✗	✗
0.117	LuFeO3	✗	✓	✓	✓
0.119	CoSe2O5	✗	✓	✓	✓

Continued on next page

index	material	inversion	antiferromagnetic	total	sublattice
0.121	Li2Co(SO4)2	✓	✗	✗	✗
0.127	Dy	✓	✓	✗	✓
0.129	Cu3Mo2O9	✗	✓	✓	✓
0.130	Cu3Mo2O9	✗	✓	✓	✓
0.132	Mn(C2N3)2	✓	✗	✗	✗
0.133	Ni3B7ClO13	✗	✓	✓	✓
0.134	Mn3B7IO13	✗	✓	✓	✓
0.135	Ni3B7BrO13	✗	✓	✓	✓
0.136	Co3B7BrO13	✗	✓	✓	✓
0.140	Lu(Fe2Ge)2	✗	✓	✓	✓
0.141	Tb5Ge4	✗	✓	✓	✓
0.145	Co3TeO6	✗	✓	✓	✓
0.150	NiS2	✓	✓	✗	✗
0.152	LiFePO4	✗	✓	✓	✓
0.157	Yb2Sn2O7	✓	✗	✗	✗
0.158	Yb2Ti2O7	✓	✗	✗	✗
0.159	DyCoO3	✗	✓	✓	✓
0.160	TbCoO3	✗	✓	✓	✓
0.164	Y2MnCoO6	✓	✗	✗	✗
0.165	SrMnVHO5	✗	✗	✓	✓
0.167	Nd3Mg2Sb3O14	✓	✗	✗	✗
0.168	Fe2H4NF6	✓	✓	✗	✓
0.169	U3As4	✗	✗	✗	✓
0.170	U3P4	✗	✗	✗	✓
0.171	DyScO3	✗	✓	✓	✓
0.175	Ca2CoSi2O7	✗	✗	✓	✓
0.177	Mn3GaN	✓	✓	✗	✗
0.179	FeH10N2Cl5O	✗	✗	✓	✓
0.182	KCrF4	✗	✓	✓	✓
0.184	Nd5Si4	✗	✗	✓	✓
0.185	Nd5Ge4	✓	✗	✗	✓
0.188	CeMnAsO	✗	✓	✓	✓
0.191	BaCuF4	✗	✗	✓	✓
0.192	RbFe2F6	✓	✓	✗	✓
0.196	Nb2Co4O9	✗	✓	✓	✓
0.197	Nb2Co4O9	✗	✓	✓	✓
0.199	Mn3Sn	✓	✓	✗	✓
0.200	Mn3Sn	✓	✓	✗	✓
0.203	Mn3Ge	✓	✗	✗	✓
0.204	Ca2MnReO6	✓	✗	✗	✗
0.207	Tl5(Fe4Se5)2	✓	✓	✗	✓
0.210	Sr2CoOsO6	✓	✓	✗	✗
0.218	Co2SiO4	✓	✓	✗	✓
0.219	Co2SiO4	✓	✓	✗	✓
0.220	Mn2SiO4	✓	✗	✗	✓
0.221	Fe2SiO4	✓	✓	✗	✓
0.236	Ca(Al2Fe)4	✓	✓	✗	✗
0.237	Er2Sn2O7	✓	✓	✗	✗
0.238	Er2Sn2O7	✓	✓	✗	✗
0.240	Er2Cu2O5	✗	✓	✓	✓
0.242	Tm2Cu2O5	✗	✗	✓	✓
0.253	Cs2FeH2Cl5O	✗	✗	✓	✓
0.262	FeCoPO5	✓	✓	✗	✓
0.268	Tb2MnNi	✗	✗	✓	✓
0.269	Tb2MnNi	✓	✗	✗	✓
0.273	Mn3ZnN	✓	✓	✗	✗
0.279	Mn3As	✓	✓	✗	✓
0.280	Mn3As	✓	✓	✗	✓
0.281	V2Co2O7	✗	✓	✓	✓
0.287	SrVCoHO5	✗	✓	✓	✓

Continued on next page

index	material	inversion	antiferromagnetic	total	sublattice
0.288	NdMnO3	✓	✗	✗	✗
0.289	NdMnO3	✓	✗	✗	✓
0.292	NiTe2O5	✓	✓	✗	✓
0.294	Cu4H6BrO6F	✓	✗	✗	✓
0.295	Cu2H3ClO3	✓	✓	✗	✓
0.296	Cu2ClO3	✓	✗	✗	✓
0.298	BaNa2V2FeO8	✓	✗	✗	✗
0.311	CoGeO3	✗	✓	✓	✓
0.313	MnGeO3	✗	✓	✓	✓
0.314	ZrCo2(GeO3)4	✓	✗	✗	✗
0.316	DyCrWO6	✗	✓	✓	✓
0.318	Tm2MnCoO6	✓	✗	✗	✓
0.319	Tm2MnCoO6	✓	✗	✗	✓
0.320	U2InPd2	✗	✓	✓	✓
0.321	U2SnPd2	✗	✓	✓	✓
0.324	Yb2CdS4	✓	✓	✗	✗
0.326	Nd2Sn2O7	✓	✓	✗	✗
0.328	KMnF4	✓	✓	✗	✗
0.337	NdFeO3	✓	✓	✗	✓
0.339	Nd2Hf2O7	✓	✓	✗	✗
0.340	Nd2Zr2O7	✓	✓	✗	✗
0.342	Tb3Ge5	✗	✓	✓	✓
0.347	Er2ReC2	✗	✓	✓	✓
0.349	Nd2NiO4	✓	✗	✗	✓
0.350	TbAlO3	✗	✓	✓	✓
0.352	TbFeO3	✓	✗	✗	✓
0.353	TbFeO3	✗	✓	✓	✓
0.357	CaFe5O7	✓	✗	✗	✓
0.368	CoH9C4NO6	✓	✗	✗	✗
0.369	CoH9C4NO6	✓	✗	✗	✗
0.370	NdMnO3	✓	✗	✗	✗
0.371	NdMnO3	✓	✗	✗	✓
0.375	La2CoIrO6	✓	✗	✗	✗
0.377	Mn3Ge	✓	✓	✗	✓
0.385	LiCoPO4	✗	✓	✓	✓
0.387	Fe3BO5	✗	✗	✓	✓
0.388	Al2Co3(SiO4)3	✗	✓	✓	✓
0.394	CdCu2(BO3)2	✗	✓	✓	✓
0.396	MnGaPt	✓	✗	✗	✗
0.398	Ca2RuO4	✓	✓	✗	✗
0.407	NdSi	✓	✗	✗	✓
0.408	PrSi	✓	✗	✗	✓
0.409	TmNi	✓	✗	✗	✓
0.411	Tb5Ge4	✗	✓	✓	✓
0.412	Tb5Ge4	✗	✓	✓	✓
0.422	EuMnSb2	✗	✓	✓	✓
0.424	EuMnSb2	✗	✓	✓	✓
0.425	Na2CoP2O7	✗	✓	✓	✓
0.430	Yb3Pt4	✗	✓	✓	✓
0.431	Cu(BO2)2	✗	✓	✓	✓
0.437	Ho3NiGe2	✓	✗	✗	✓
0.438	Pr3CoGe2	✓	✗	✗	✓
0.440	SrCu(TeO3)2	✗	✓	✓	✓
0.441	Nb2Fe4O9	✗	✓	✓	✓
0.446	MnCoGe	✓	✗	✗	✓
0.449	Tb2Pt	✓	✗	✗	✓
0.450	Nd5Ge4	✓	✗	✗	✓
0.473	La(MnSi)2	✗	✗	✗	✗
0.478	SmCrO3	✓	✓	✗	✓
0.479	SmCrO3	✓	✗	✗	✓

Continued on next page

index	material	inversion	antiferromagnetic	total	sublattice
0.480	HoNi	✓	✗	✗	✓
0.481	HoNi	✓	✗	✗	✓
0.487	Er(CrSi)2	✗	✗	✗	✗
0.488	YbMnO3	✗	✓	✓	✓
0.489	YbMnO3	✗	✓	✓	✓
0.490	YbMnO3	✗	✗	✓	✓
0.491	NdB4	✗	✓	✓	✓
0.492	NdB4	✗	✓	✓	✓
0.495	La(MnSi)2	✗	✗	✗	✗
0.496	La(MnSi)2	✗	✗	✗	✗
0.497	La(MnSi)2	✗	✗	✗	✗
0.506	Cs2Cu3SnF12	✓	✓	✗	✓
0.511	Ta2Co4O9	✗	✓	✓	✓
0.520	TbCoO3	✗	✓	✓	✓
0.521	DyCoO3	✗	✓	✓	✓
0.529	Nb2Co4O9	✗	✓	✓	✓
0.530	SrCu(TeO3)2	✗	✓	✓	✓
0.544	Mn2FeReO6	✓	✗	✗	✓
0.545	Mn2FeReO6	✓	✗	✗	✓
0.550	Mn3ReO6	✓	✓	✗	✓
0.551	Mn3ReO6	✓	✓	✗	✓
0.552	Mn(PbO2)2	✗	✓	✓	✓
0.564	U2Si5Rh3	✓	✓	✗	✓
0.565	Ce2Ni3Ge5	✓	✓	✗	✓
0.571	CoSO4	✓	✓	✗	✗
0.572	Na2CrNiF7	✓	✗	✗	✗
0.573	Na2CrNiF7	✓	✗	✗	✗
0.574	MnFeH4O2F5	✗	✗	✓	✓
0.576	Cr2F5	✓	✗	✗	✗
0.577	BaMnFeF7	✓	✗	✗	✓
0.578	BaNaFe2F9	✓	✗	✗	✓
0.579	Na2FeNiF7	✓	✗	✗	✗
0.580	Na2FeNiF7	✓	✗	✗	✗
0.583	Fe2H4O2F5	✓	✗	✗	✗
0.584	Fe2H4O2F5	✓	✗	✗	✗
0.588	PrCrO3	✓	✗	✗	✓
0.589	NdCrO3	✓	✓	✗	✓
0.590	ErCrO3	✓	✓	✗	✓
0.612	Cu2SO5	✓	✗	✗	✓
0.625	U2InPd2	✗	✓	✓	✓
0.641	Mn3Ga	✓	✗	✗	✗
0.652	HoMnO3	✗	✓	✓	✓
0.656	Nd(MnGe)2	✗	✗	✗	✗
0.657	Pr(MnGe)2	✗	✗	✗	✗
0.658	BaCu(TeO3)2	✗	✓	✓	✓
0.663	Mn3Sn2	✓	✗	✗	✓
0.664	Mn3Sn2	✓	✗	✗	✓
0.684	TbPt	✓	✗	✗	✓
0.685	ErPt	✓	✗	✗	✓
0.686	HoPt	✓	✗	✗	✓
0.687	DyPt	✓	✗	✗	✓
0.688	TmPt	✓	✗	✗	✓
0.697	SmCrO3	✓	✗	✗	✓
0.698	SmCrO3	✓	✓	✗	✓
0.707	Tb2(Ga3Ir)3	✓	✗	✗	✓
0.715	HoCrWO6	✗	✓	✓	✓
0.716	HoCrWO6	✗	✓	✓	✓
0.724	BaCoSiO4	✗	✓	✓	✓
0.727	CsMn2F6	✓	✗	✗	✓
0.740	Dy3Ga5O12	✓	✓	✗	✓

Continued on next page

index	material	inversion	antiferromagnetic	total	sublattice
0.741	Er3Ga5O12	✓	✓	✗	✓
0.743	Ho3Al5O12	✓	✓	✗	✓
0.744	Tb3Al5O12	✓	✓	✗	✓
0.745	Ho3Ga5O12	✓	✓	✗	✓
0.746	Tb3Ga5O12	✓	✓	✗	✓
0.756	V4GaS8	✗	✗	✓	✓
0.761	SrFe2Se2O	✗	✓	✓	✓
0.762	SrFe2S2O	✗	✓	✓	✓
0.763	Mn5P4(HO2)10	✓	✗	✗	✓
0.764	Mn5P4(HO2)10	✓	✗	✗	✓
0.765	Mn5P4(HO2)10	✓	✗	✗	✓
0.768	SrMnSb2	✗	✗	✓	✓
0.773	NdMnSi2	✓	✗	✗	✓
0.782	NdScO3	✗	✓	✓	✓
0.783	NdInO3	✗	✓	✓	✓
0.785	NdVO3	✓	✓	✗	✓
0.788	YVO3	✓	✓	✗	✗
0.803	MnNbP	✗	✓	✓	✓
0.809	Fe2WO6	✗	✓	✓	✓
0.810	Fe2WO6	✓	✗	✗	✓
0.812	Fe2WO6	✗	✓	✓	✓
0.818	Ta2MnO6	✗	✓	✓	✓
0.819	MnNb2O6	✗	✓	✓	✓
0.831	BaCaFe4O7	✗	✗	✓	✓
1.0.4	CsNiCl3	✗	✓	✓	✓
1.0.8	Ba3MnNb2O9	✗	✓	✗	✗
1.0.14	CsFeCl3	✗	✓	✗	✗
1.0.22	K2Mn3V2CO11	✗	✓	✗	✓
1.0.24	ThMn2	✗	✓	✗	✓
1.0.31	Eu(InAs)2	✗	✓	✓	✓
1.0.32	Eu(InAs)2	✗	✓	✓	✓
1.0.33	FeF3	✓	✓	✗	✗
1.0.35	CsMnBr3	✗	✓	✗	✗
1.0.40	RbFeCl3	✗	✓	✗	✗
1.0.41	RbNiCl3	✗	✓	✗	✗
1.0.42	CsNiCl3	✗	✓	✗	✗
1.0.44	Ba3CoSb2O9	✗	✓	✗	✗
1.0.45	Ba3CoSb2O9	✗	✓	✗	✗
1.0.46	Ba3MnSb2O9	✗	✓	✓	✓
1.0.49	BaCoSiO4	✗	✓	✓	✓
1.0.50	CoGeO3	✓	✗	✗	✓
1.0.52	Tb7Ag27	✗	✓	✓	✓
1.2	CuSe2O5	✓	✓	✗	✗
1.13	Ba3Nb2NiO9	✗	✓	✗	✗
1.14	BaHo2NiO5	✓	✓	✗	✓
1.15	BaHo2NiO5	✓	✓	✗	✓
1.19	PrMn2O5	✗	✓	✓	✓
1.25	KFe3(SO7)2	✓	✓	✗	✗
1.34	HoGeAu	✗	✓	✗	✓
1.36	BaDy2NiO5	✓	✓	✗	✓
1.38	NaNd2OsO6	✓	✓	✗	✓
1.39	LiFe(GeO3)2	✓	✓	✗	✓
1.44	NdNiO3	✗	✓	✗	✓
1.48	HoNiO3	✗	✓	✓	✓
1.51	Cs2CoCl4	✗	✓	✓	✓
1.53	BaEr2NiO5	✓	✓	✗	✓
1.54	GdMn2O5	✗	✓	✓	✓
1.67	TmInPt	✗	✓	✓	✓
1.68	NaNdFeWO6	✗	✓	✓	✓
1.73	CaV2O4	✓	✓	✗	✓

Continued on next page

index	material	inversion	antiferromagnetic	total	sublattice
1.74	Mn2BiO5	✗	✓	✓	✓
1.75	Mn2BiO5	✗	✓	✓	✓
1.76	DyMn2O5	✗	✓	✓	✓
1.77	Sr2IrO4	✓	✓	✗	✓
1.83	Ba2Fe2O5	✓	✓	✗	✓
1.85	Mn	✗	✓	✓	✓
1.88	Mn5Si3	✓	✓	✗	✓
1.89	DyFe3(BO3)4	✗	✓	✓	✓
1.92	HoFe3(BO3)4	✗	✓	✓	✓
1.93	HoFe3(BO3)4	✗	✓	✗	✓
1.95	BaNd2O4	✓	✓	✗	✓
1.96	BaNd2O4	✓	✓	✗	✓
1.98	Dy(Fe2Ge)2	✗	✓	✓	✓
1.99	CsCoH4Cl3O2	✓	✓	✗	✓
1.102	U2InNi2	✓	✓	✗	✓
1.108	TbMn2O5	✗	✓	✓	✓
1.109	HoMn2O5	✗	✓	✓	✓
1.114	Ca4IrO6	✓	✓	✗	✗
1.115	Dy3(Al3Ru)4	✓	✓	✗	✓
1.116	MnVAgO4	✓	✓	✗	✗
1.117	NaFePO4	✓	✓	✗	✗
1.118	GdPO4	✓	✓	✗	✓
1.122	Cu3BiSe2BrO8	✓	✓	✗	✓
1.123	YCu3Se2ClO8	✓	✓	✗	✓
1.124	BaYFe4O7	✗	✓	✓	✓
1.126	NaCoSO4F	✓	✓	✗	✗
1.127	NiBiPO5	✓	✓	✗	✓
1.128	CoBiPO5	✓	✓	✗	✓
1.129	Fe3AgH6(SO7)2	✓	✓	✗	✗
1.133	Cu(SbO3)2	✓	✓	✗	✗
1.135	Co2H4C8O11	✓	✓	✗	✓
1.138	MgV2O4	✗	✓	✓	✓
1.147	Li2Fe(SO4)2	✓	✓	✗	✗
1.161	PrFe3(BO3)4	✗	✓	✓	✓
1.167	NiS2	✓	✓	✗	✗
1.170	Tm5(In2Ni)2	✗	✓	✓	✓
1.185	Cu2GeO4	✗	✓	✗	✓
1.196	MnV2O6	✓	✓	✗	✓
1.197	Fe4Si2Sn7O16	✓	✓	✗	✗
1.201	Cr2ReO8	✓	✓	✗	✓
1.202	CrReO4	✓	✓	✗	✓
1.203	Si(NiO2)2	✓	✓	✗	✓
1.204	Si(NiO2)2	✓	✓	✗	✓
1.207	U2SnRh2	✓	✓	✗	✓
1.216	BaNd2NiO5	✓	✓	✗	✓
1.217	BaTb2NiO5	✓	✓	✗	✓
1.218	BaTm2NiO5	✓	✓	✗	✓
1.224	Nb2CoO6	✗	✓	✓	✓
1.226	CeCo2(GeO3)4	✓	✓	✗	✗
1.227	Ca2Cr2O5	✗	✓	✓	✓
1.235	BaTiCu4P4O17	✗	✓	✓	✓
1.237	VCl2	✗	✓	✗	✗
1.238	VBr2	✗	✓	✗	✗
1.266	SmFe3(BO3)4	✗	✓	✗	✓
1.267	Dy2(Al3Co)3	✗	✓	✓	✓
1.272	CeNiAsO	✗	✓	✓	✓
1.274	DyFeWO6	✗	✓	✓	✓
1.279	Ho2Cu2O5	✗	✓	✓	✓
1.280	Yb2Cu2O5	✗	✓	✓	✓
1.299	GdMn2	✗	✓	✓	✓

Continued on next page

index	material	inversion	antiferromagnetic	total	sublattice
1.300	GdMn2	✗	✓	✓	✓
1.301	MnBiTeO6	✓	✓	✗	✓
1.302	Ba2CoO4	✓	✓	✗	✓
1.303	Dy3(Al3Ru)4	✓	✓	✗	✓
1.306	BaNa2MnV2O8	✓	✓	✗	✗
1.307	Mn5Si3	✗	✓	✓	✓
1.323	CoGeO3	✓	✓	✗	✓
1.324	DyMn2O5	✗	✓	✓	✓
1.326	PrMn2O5	✓	✓	✗	✓
1.327	LaMn2O5	✓	✓	✗	✓
1.328	Yb2MnCoO6	✗	✓	✓	✓
1.330	Lu2MnCoO6	✗	✓	✓	✓
1.338	U2InNi2	✓	✓	✗	✓
1.340	LuMnO3	✗	✓	✗	✓
1.342	Co3(PO4)2	✓	✓	✗	✓
1.343	Ba2Co9O14	✓	✓	✗	✓
1.345	NaMnF4	✓	✓	✗	✗
1.350	BaNd2CoO5	✓	✓	✗	✓
1.352	Ba2Ni2ClF7	✓	✓	✗	✓
1.357	Ho3Ge4	✓	✓	✗	✓
1.359	Dy3Ge4	✓	✓	✗	✓
1.362	Er3Ge4	✓	✓	✗	✓
1.380	Sr2FeClO3	✗	✓	✗	✓
1.381	Sr2FeBrO3	✗	✓	✗	✓
1.382	Ca2FeClO3	✗	✓	✗	✓
1.383	Ca2FeBrO3	✗	✓	✗	✓
1.385	Sr2FeO3F	✗	✓	✗	✓
1.386	Sr2FeO3F	✗	✓	✗	✓
1.387	Sr2FeO3F	✗	✓	✗	✓
1.418	Cu4O3	✗	✓	✗	✓
1.431	Ca2Mn3O8	✓	✓	✗	✓
1.441	NaFe3H6(SO7)2	✓	✓	✗	✗
1.443	BaGd2CuO5	✗	✓	✓	✓
1.444	Er2Pt	✗	✓	✓	✓
1.455	Mn6Si7Ni16	✓	✓	✗	✓
1.456	Sr2Cu3(SeO)2	✗	✓	✗	✗
1.476	Ba2CoO4	✓	✓	✗	✓
1.477	Ba2CoO4	✓	✓	✗	✓
1.484	Li2MnGeO4	✗	✓	✓	✓
1.498	CuSi(HO2)2	✓	✓	✗	✓
1.499	CsFe(MoO4)2	✗	✓	✓	✓
1.519	CoSO4	✓	✓	✗	✗
1.524	MnInO3	✗	✓	✗	✓
1.525	MnInO3	✗	✓	✗	✓
1.528	Fe4Bi2O9	✓	✓	✗	✓
1.533	TbC2	✗	✓	✓	✓
1.549	U2InNi2	✓	✓	✗	✓
1.577	SrNd2O4	✓	✓	✗	✓
1.584	PrFeAsO	✓	✓	✗	✓
1.587	NdFeAsO	✓	✓	✗	✓
1.595	CaCoSO	✗	✓	✓	✓
1.599	DyMn2O5	✗	✓	✓	✓
1.622	CoGeO3	✓	✓	✗	✓
1.649	Sr3ZnIrO6	✓	✓	✗	✗
1.651	BaHo2CuO5	✓	✓	✗	✓
1.657	LuNiO3	✗	✓	✓	✓
1.660	FeSb6(Pb2S7)2	✓	✓	✗	✗
1.661	La2NiIrO6	✓	✓	✗	✗
1.662	La2NiIrO6	✓	✓	✗	✗
2.2	Sr2Fe2S2OF2	✓	✓	✗	✓

Continued on next page

index	material	inversion	antiferromagnetic	total	sublattice
2.3	HoNiO3	✗	✓	✓	✓
2.5	Mn3CuN	✓	✗	✗	✓
2.6	Nd2CuO4	✓	✓	✗	✗
2.7	Sm2CuO4	✓	✓	✗	✗
2.8	SrHo2O4	✗	✓	✓	✓
2.9	Ca3CuNi2(PO4)4	✓	✓	✗	✓
2.10	HoP	✓	✗	✗	✗
2.11	TbMg	✓	✗	✗	✗
2.12	TbMg	✓	✗	✗	✗
2.13	UP	✓	✓	✗	✗
2.14	NdMg	✓	✓	✗	✗
2.18	Sc2MnNiO6	✓	✓	✗	✓
2.19	Mn3ZnC	✓	✗	✗	✗
2.20	UAs	✓	✓	✗	✓
2.21	TbHO2	✗	✓	✓	✓
2.22	Ta2FeO6	✓	✓	✗	✗
2.23	Sr2CoAg2(SeO)2	✓	✓	✗	✗
2.24	Ba2CoAg2(SeO)2	✓	✓	✗	✗
2.27	Sr2Mn3(SbO)2	✗	✓	✗	✓
2.28	NpGa5Ni	✓	✗	✗	✗
2.29	Mn3O4	✓	✗	✗	✓
2.31	Mn3ZnN	✓	✓	✗	✓
2.33	Na2Mn3Se4	✓	✓	✗	✓
2.35	CrSe	✗	✓	✗	✗
2.48	Pr2CuO4	✓	✓	✗	✗
2.49	La2Fe2Se2O3	✓	✓	✗	✓
2.51	EuMnBi2	✗	✗	✗	✓
2.52	Mn3O4	✗	✗	✓	✓
2.53	Ba2Mn3(SbO)2	✗	✓	✗	✓
2.54	Sr2Cr3(AsO)2	✓	✓	✗	✓
2.55	Sr2Fe3Se2O3	✗	✓	✓	✓
2.56	La2Fe2S2O3	✓	✓	✗	✓
2.57	Tb(MnSi)2	✓	✗	✗	✓
2.60	Nd(MnSi)2	✓	✗	✗	✓
2.61	Fe3H4(OF4)2	✓	✓	✗	✗
2.62	TbCrO3	✗	✓	✓	✓
2.63	DyCrO3	✓	✓	✗	✓
2.64	DyCrO3	✓	✓	✗	✓
2.66	FeSn2	✗	✓	✗	✗
2.67	FeSn2	✓	✓	✗	✗
2.68	FeGe2	✗	✓	✗	✗
2.70	GdMg	✓	✗	✗	✗
2.71	HoRh	✓	✓	✗	✗
2.72	Nb3VS6	✗	✗	✗	✓
2.73	BaNd2ZnO5	✓	✓	✗	✓
2.75	Sr2Fe3S2O3	✓	✓	✗	✓
2.76	Sr2Fe3Se2O3	✗	✓	✓	✓
2.77	Eu2CuO4	✓	✓	✗	✗
2.78	Nd2CuO4	✓	✓	✗	✗
2.79	Pr2CuO4	✓	✓	✗	✗
2.81	Er(MnSi)2	✓	✗	✗	✓
2.82	Er(MnSi)2	✓	✗	✗	✓
2.83	Er(MnGe)2	✓	✗	✗	✓
2.84	Er(MnGe)2	✓	✗	✗	✓
2.85	BaHo2CuO5	✗	✓	✓	✓
2.86	Ta2FeO6	✓	✓	✗	✗
3.1	TmAgGe	✗	✓	✓	✓
3.2	UO2	✓	✓	✗	✗
3.4	MgCr2O4	✗	✓	✗	✓
3.6	DyCu	✓	✓	✗	✗

Continued on next page

index	material	inversion	antiferromagnetic	total	sublattice
3.7	NpBi	✓	✓	✗	✗
3.8	NdZn	✓	✓	✗	✗
3.9	NpS	✓	✓	✗	✗
3.10	NpSe	✓	✓	✗	✗
3.11	NpTe	✓	✓	✗	✗
3.12	NpSb	✓	✓	✗	✗
3.13	CeB6	✓	✓	✗	✓
3.18	HoRh	✓	✓	✗	✗
3.19	CoO	✓	✓	✗	✗

[1] J. Zelezný, "symcode," (2017).
[2] J. Železný, H. Gao, A. Manchon, F. Freimuth, Y. Mokrousov, J. Zemen, J. Mašek, J. Sinova, and T. Jungwirth, Physical Review B **95**, 014403 (2017).
[3] W. F. Brinkman and R. J. Elliott, Proceedings of the Royal Society of London Series A **294**, 343 (1966).
[4] D. B. Litvin and W. Opechowski, Physica **76**, 538 (1974).
[5] S. V. Gallego, J. M. Perez-Mato, L. Elcoro, E. S. Tasci, R. M. Hanson, K. Momma, M. I. Aroyo, and G. Madariaga, J. Appl. Crystallogr. **49**, 1750 (2016).
[6] S. V. Gallego, J. M. Perez-Mato, L. Elcoro, E. S. Tasci, R. M. Hanson, M. I. Aroyo, and G. Madariaga, J. Appl. Crystallogr. **49**, 1941 (2016).
